# Labeled temperate hardwood tree stomatal image datasets from seven taxa of *Populus* and 17 hardwood species

**DOI:** 10.1038/s41597-023-02657-3

**Published:** 2024-01-02

**Authors:** Jiaxin Wang, Heidi J. Renninger, Qin Ma

**Affiliations:** 1 Department of Forestry, Forest and Wildlife Research Center, Mississippi State University, Mississippi State, 39762 USA; 2 School of Geography, Nanjing Normal University, Nanjing, 210023 China; 3 Key Laboratory of Virtual Geographic Environment (Nanjing Normal University), Ministry of Education, 210023 Nanjing, China; 4 Jiangsu Center for Collaborative Innovation in Geographical Information Resource Development and Application, 210023, Nanjing, China

**Keywords:** Forest ecology, Forestry

## Abstract

Machine learning (ML) algorithms have shown potential in automatically detecting and measuring stomata. However, ML algorithms require substantial data to efficiently train and optimize models, but their potential is restricted by the limited availability and quality of stomatal images. To overcome this obstacle, we have compiled a collection of around 11,000 unique images of temperate broadleaf angiosperm tree leaf stomata from various projects conducted between 2015 and 2022. The dataset includes over 7,000 images of 17 commonly encountered hardwood species, such as oak, maple, ash, elm, and hickory, and over 3,000 images of 55 genotypes from seven *Populus* taxa. *Inner_guard_cell_walls* and *whole_stomata* (stomatal aperture and guard cells) were labeled and had a corresponding YOLO label file that can be converted into other annotation formats. With the use of our dataset, users can (1) employ state-of-the-art machine learning models to identify, count, and quantify leaf stomata; (2) explore the diverse range of stomatal characteristics across different types of hardwood trees; and (3) develop new indices for measuring stomata.

## Background & Summary

Stomatal responses to environmental factors, such as humidity and soil moisture, are crucial for driving photosynthesis, productivity, water yield, ecohydrology, and climate forcing^1–4^. However, to fully understand these responses, we must improve our understanding of the mechanistic basis of stomatal response to environmental factors^5^. Unfortunately, current stomatal studies are limited by the laborious and time-consuming process of manually counting and measuring stomatal properties, resulting in small dataset size and image scales when observing stomata. Therefore, having large stomatal image datasets for developing fast and high-throughput methods for studying stomata is highly warranted.

The potential of artificial intelligence (AI) for developing annotated, high-throughput stomatal measuring methods is high, which could significantly enhance scientists’ ability to conduct large-scale and intensive stomatal studies. Recently, state-of-the-art machine learning algorithms, such as deep learning, specifically convolutional neural networks (CNNs), have been designed to solve complex image detection and segmentation problems, resulting in various applications tailored to specific objectives^6,7^. One of the most efficient and straightforward CNN architectures is You Only Look Once (YOLO), proposed by Redmon, *et al*.^8^. This architecture has been used for stomatal detection, counting^9–12^, and measuring^12,13^. These studies have shown the potential of using machine learning algorithms for automated stomatal detection and measurement. However, fine-tuning and improvement of machine learning-based stomatal study methods are currently limited by the small, inconsistent, and monotypic nature of stomatal image datasets, which are also poorly accessible.

Many studies have increased stomatal image datasets during machine learning training to avoid overfitting using augmentation techniques such as random translation, rotation, flipping, and zooming^9,14^. While image preprocessing techniques can increase the training sample size, model performance may still be limited due to variability in stomatal characteristics. For example, some methods trained using specific species datasets may only be sensitive to those species and cannot be generalized for other species^9^. Therefore, it is crucial to create a publicly accessible leaf stomatal image database to develop machine learning-based, state-of-the-art stomatal measuring methods to be used by ecologists, plant biologists, and ecophysiologists.

Our collection consists of around 11,000 unique images of hardwood leaf stomata collected from projects conducted between 2015 and 2022. Within the hardwood stomatal dataset, there are more than 7,000 images of 17 common hardwood species, such as oak, maple, ash, elm, and hickory. Additionally, the dataset contains over 3,000 images of 55 genotypes from seven *Populus* taxa (Tables 1, 2). We labeled *inner_guard_cell_walls* as “0”, *whole_stomata* (stomatal aperture and guard cells) as “1” and created a YOLO label file for each image. These images and corresponding labels are freely accessible, making it easier to train machine-learning models and analyze leaf stomatal traits. With the help of our dataset, individuals can: (1) utilize cutting-edge machine learning models to train for high-throughput detection, counting, and measurement of leaf stomata of temperate hardwood trees; (2) investigate the diversity in stomatal characteristics across various types of hardwood trees; (3) develop novel indices for measuring stomata.

**Table 1:** Plant species used for this study (checked based on Integrated Taxonomic Information System (ITIS, www.itis.gov)).

Datasets	Taxa	Common names	Scientific names and authorities	Genotypes
Hardwoods	NA	Ash	*Fraxinus* L.	NA
	NA	Black gum	*Nyssa sylvatica* Marshall	NA
	NA	Deerberry	*Vaccinium stamineum* Linneaus	NA
	NA	Leatherwood	*Dirca palustris* L.	NA
	NA	American elm	*Ulmus americana* Planch	NA
	NA	Cherrybark oak	*Quercus pagoda* Raf.	NA
	NA	Red maple	*Acer rubrum* L.	NA
	NA	Nuttall oak	*Quercus texana* Buckley	NA
	NA	Post oak	*Quercus stellata* Wangenh.	NA
	NA	Shagbark hickory	*Carya ovata* (Mill.) K. Koch	NA
	NA	Shumard oak	*Quercus shumardii* Buckley	NA
	NA	Swamp chestnut oak	*Quercus michauxii* Nutt.	NA
	NA	Water oak	*Quercus nigra* L.	NA
	NA	Willow	*Salix* spp.	NA
	NA	Willow oak	*Quercus phellos* L.	NA
	NA	Winged elm	*Ulmus alata* Michx.	NA
*Populus* I	D × D	Eastern cottonwood	*Populus deltoides* × *Populus deltoides*	106B-1
	D × D	Eastern cottonwood	*Populus deltoides* × *Populus deltoides*	110412
	D × D	Eastern cottonwood	*Populus deltoides* × *Populus deltoides*	111733
	D × D	Eastern cottonwood	*Populus deltoides* × *Populus deltoides*	112107
	D × D	Eastern cottonwood	*Populus deltoides* × *Populus deltoides*	113B-3
	D × D	Eastern cottonwood	*Populus deltoides* × *Populus deltoides*	120-4
	D × D	Eastern cottonwood	*Populus deltoides* × *Populus deltoides*	19
	D × D	Eastern cottonwood	*Populus deltoides* × *Populus deltoides*	3-1
	D × D	Eastern cottonwood	*Populus deltoides* × *Populus deltoides*	47-5
	D × D	Eastern cottonwood	*Populus deltoides* × *Populus deltoides*	6-4
	D × D	Eastern cottonwood	*Populus deltoides* × *Populus deltoides*	6-5
	D × D	Eastern cottonwood	*Populus deltoides* × *Populus deltoides*	S7C2
	D × D	Eastern cottonwood	*Populus deltoides* × *Populus deltoides*	S7C20
	D × D	Eastern cottonwood	*Populus deltoides* × *Populus deltoides*	ST66
	D × D	Eastern cottonwood	*Populus deltoides* × *Populus deltoides*	ST75

**Table 2:** Hybrid poplars used for this study.

Datasets	Taxa	Common names	Scientific names and authorities	Genotypes
*Populus* II	D × M	Poplar hybrid	*Populus deltoides* × *Populus maximowiczii*	11666
	D × M	Poplar hybrid	*Populus deltoides* × *Populus maximowiczii*	11732
	D × M	Poplar hybrid	*Populus deltoides* × *Populus maximowiczii*	13724
	D × M	Poplar hybrid	*Populus deltoides* × *Populus maximowiczii*	13725
	D × M	Poplar hybrid	*Populus deltoides* × *Populus maximowiczii*	14490
	D × M	Poplar hybrid	*Populus deltoides* × *Populus maximowiczii*	14507
	D × M	Poplar hybrid	*Populus deltoides* × *Populus maximowiczii*	14508
	D × M	Poplar hybrid	*Populus deltoides* × *Populus maximowiczii*	24033
	D × M	Poplar hybrid	*Populus deltoides* × *Populus maximowiczii*	24159
	D × M	Poplar hybrid	*Populus deltoides* × *Populus maximowiczii*	29310
	D × M	Poplar hybrid	*Populus deltoides* × *Populus maximowiczii*	6323
	D × M	Poplar hybrid	*Populus deltoides* × *Populus maximowiczii*	7388
	D × M	Poplar hybrid	*Populus deltoides* × *Populus maximowiczii*	8015
	D × M	Poplar hybrid	*Populus deltoides* × *Populus maximowiczii*	8019
	D × M	Poplar hybrid	*Populus deltoides* × *Populus maximowiczii*	9189
	D × M	Poplar hybrid	*Populus deltoides* × *Populus maximowiczii*	9225
	D × M	Poplar hybrid	*Populus deltoides* × *Populus maximowiczii*	9671
	D × M	Poplar hybrid	*Populus deltoides* × *Populus maximowiczii*	9707
	D × N	Poplar hybrid	*Populus deltoides* × *Populus nigra*	11789
	D × N	Poplar hybrid	*Populus deltoides* × *Populus nigra*	11797
	D × N	Poplar hybrid	*Populus deltoides* × *Populus nigra*	11802
	D × N	Poplar hybrid	*Populus deltoides* × *Populus nigra*	11822
	D × N	Poplar hybrid	*Populus deltoides* × *Populus nigra*	11840
	D × N	Poplar hybrid	*Populus deltoides* × *Populus nigra*	11859
	D × N	Poplar hybrid	*Populus deltoides* × *Populus nigra*	11867
	D × N	Poplar hybrid	*Populus deltoides* × *Populus nigra*	14278
	D × N	Poplar hybrid	*Populus deltoides* × *Populus nigra*	14340
	D × N	Poplar hybrid	*Populus deltoides* × *Populus nigra*	433
	D × N × M	Poplar hybrid	*Populus deltoides* × *nigra* × *maximowiczii*	24250
	D × T	Poplar hybrid	*Populus deltoides* × *Populus trichocarpa*	10016
	D × T	Poplar hybrid	*Populus deltoides* × *Populus trichocarpa*	7903
	D × T	Poplar hybrid	*Populus deltoides* × *Populus trichocarpa*	7938
	D × T	Poplar hybrid	*Populus deltoides* × *Populus trichocarpa*	8717
	D × T	Poplar hybrid	*Populus deltoides* × *Populus trichocarpa*	8729
	T × D	Poplar hybrid	*Populus trichocarpa* × *Populus deltoides*	9755
	T × M	Poplar hybrid	*Populus trichocarpa* × *Populus maximowiczii*	24301
	T × M	Poplar hybrid	*Populus trichocarpa* × *Populus maximowiczii*	24326
	T × M	Poplar hybrid	*Populus trichocarpa* × *Populus maximowiczii*	24340
	T × M	Poplar hybrid	*Populus trichocarpa* × *Populus maximowiczii*	29270
	T × M	Poplar hybrid	*Populus trichocarpa* × *Populus maximowiczii*	24245

## Methods

### Leaves and micrographs collection

The study utilized stomatal images from two datasets: Hardwood and *Populus* spp., acquired from 2015 to 2022. The Hardwood dataset contained 16 species, including American elm (*Ulmus americana* Planch), cherrybark oak (*Quercus pagoda* Raf.), Nuttall oak (*Quercus texana* Buckley), shagbark hickory (*Carya ovata* (Mill.) K. Koch), Shumard oak (*Quercus shumardii* Buckley), swamp chestnut oak (*Quercus michauxii* Nutt.), water oak (*Quercus nigra* L.), willow oak (*Quercus phellos* L.), ash (*Fraxinus* L.), black gum (*Nyssa sylvatica* Marshall), deerberry (*Vaccinium stamineum* Linneaus), leatherwood (*Dirca palustris* L.), red maple (*Acer rubrum* L.), post oak (*Quercus stellata* Wangenh.), willow (*Salix* spp.), and winged elm (*Ulmus alata* Michx.), with the age of seedlings ranging from 1–3 years for Nuttall oak, water oak, and Shumard oak, and 30–50 years for the rest. Using a compound light microscope (Olympus, Tokyo, Japan) equipped with a digital microscope camera (MU300, AmScope, USA) with a 5 mm lens and a fixed microscope adapter (FMA050, AmScope), over 10,000 stomatal images were captured. The *Populus* dataset consisted of over 3,000 images from 55 genotypes of seven taxa of hybrid poplar and eastern cottonwood (*Populus deltoides*), which were 4 to 5 years old. Detailed taxa and genotype information are shown in Tables 1 and 2.

Between June and August 2020 to 2022, we selected trees and measured their photosynthetic CO_2_ response curves (AC/_i_), after which we collected one fully expanded, fresh leaf from each tree. The leaves were placed in labeled plastic bags and kept in a cooler for transportation to the laboratory, where they were stored in a 4 °C refrigerator. Following the method described by Hilu and Randall^15^, we prepared the leaves for stomatal peels by drying any moisture on the leaves surface with paper towels and applying clear nail polish to 4–6 locations on the abaxial epidermis of the leaves. After allowing the nail polish to dry for approximately 5–8 minutes, we removed it from the leaves and placed it on pre-cleaned microscope slides, covering it with one or two coverslips. We used a 10X upper eyepiece and either an X20 or X40 magnification lens to capture three to ten images per leaf.

### Annotation process

We used manual and pre-trained model labeling methods to process image labels. Specifically, we manually labeled 1,000 images, 300 from *Populus* and 700 from other hardwood species, to train a YOLO model for detecting and measuring *inner_guard_cell_walls* and *whole_stomata*. The StoManager1, which incorporates our trained model, has been made publicly available on Zenodo^13,16^. It has a user-friendly, Graphical User Interface (GUI) version designed for Windows-based systems. We used it to automatically label *inner_guard_cell_walls* and *whole_stomata* while exporting the label coordinates to YOLO Darknet format files. It is possible to convert our YOLO Darknet format labeling files into various other annotation formats, including Pascal VOC. Users who require labeling annotations in the Pascal VOC format can utilize online conversion tools, such as Roboflow’s public workspace and open-source GitHub repositories^17,18^.

The typical format for saving YOLO annotations is a.txt file with five columns containing information about the classes (0, 1) and four variables: x_center, y_center, width, and height of the bounding boxes. The x_center and y_center are expressed as normalized coordinates that correspond to the center of the bounding box, while width and height are normalized values that represent the relative width and height of the box concerning the dimensions of the image. Since StoManager1 exported annotations were structured slightly differently from YOLO annotations, we reformatted them in R and the code is publicly available on a GitHub repository (https://github.com/JiaxinWang123/ScientificData_Labeled_Hardwood_Images).

### Label quality check

Labels created by StoManager1 were manually reviewed and adjusted using LabelImg (https://github.com/heartexlabs/labelImg) as necessary. After reviewing and modifying the labels, a subset of images was randomly chosen and used to train YOLO models for detecting the labeled classes, which included *inner_guard_cell_walls* and *whole_stomata*. To verify the accuracy of the annotations, a random selection of labeled images was split and used to train YOLOv7 and YOLOv8 models.

## Data Records

The dataset contains original images, labels, and data records available to the public on figshare^19^ and Zenodo^20^. The data records are presented in a table with 10,715 observations and seven variables. Each observation in the table corresponds to a single image, and each variable represents a column that describes the image name, species name, scientific name, magnification, width, height, and resolution (pixels per 0.1 mm line).

**Fig. 1:**
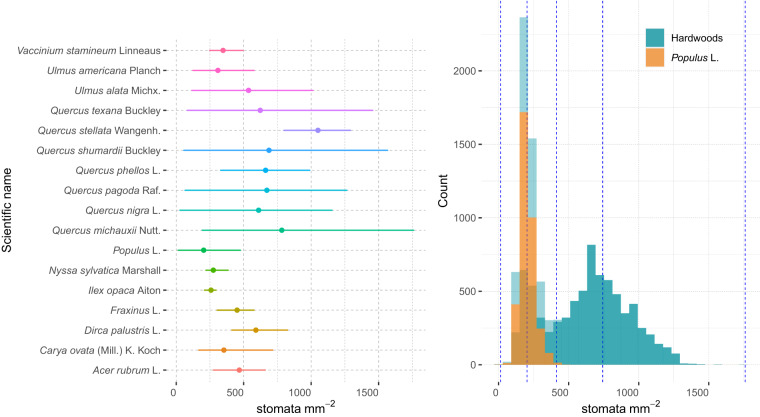
(**a**) The number of stomata per image of the 17 hardwood species in the dataset, (**b**) histogram of the number of stomata across Hardwood and *Populus* datasets. Dots in plot (**a**) indicate the mean of the stomatal density and the lines represent the range of the stomatal density. Blue dotted lines represent the percentage quantiles.

**Fig. 2:**
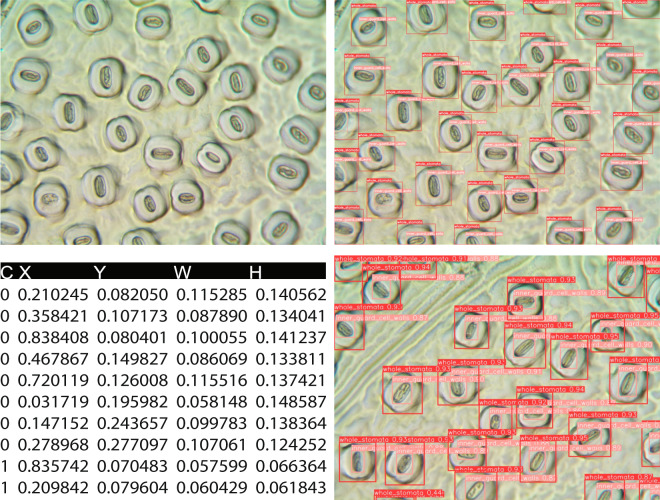
Original and annotated leaf stomatal images and the label file structure. C, X, Y, W, H represent class, x_center, y_center, width, and height of the bounding boxes, respectively. The x_center and y_center are expressed as normalized coordinates that correspond to the center of the bounding box, while width and height are normalized values that represent the relative width and height of the box concerning the dimensions of the image. Note that “C, X, Y, W, H” do not exist in label files, and we used these headings for explanation.

Every image in the dataset has a distinct file name and a corresponding label file, which contains information about classes, coordinates, width, and height. These values are expressed as ratios to the image’s width and height and pertain to the bounding boxes of *inner_guard_cell_walls* and *whole_stomata*. Figures 1, 2, and Table 3 provide more comprehensive details regarding the original images, labels, and data records. It is essential to note that magnification, width, height, and resolution are crucial variables for studying leaf stomatal area, stomatal density, and stomatal area variance because they determine the scale of stomatal observation and measurement.

**Table 3:** Data record structure.

FileName	Species	ScientificName	Magnification	Width	Height	Resolution*
STMHD0806	American elm	*Ulmus americana* Planch	200	1024	768	239
STMHD0884	American Holly	*Ilex opaca* Aiton	200	2048	1536	476
STMHD3585	Ash	*Fraxinus* L.	400	2048	1536	930
STMHD6460	Black gum	*Nyssa sylvatica* Marshall	400	2048	1536	930
STMHD0984	Cherrybark oak	*Quercus pagoda* Raf.	200	1024	768	239
STMHD3932	Deerberry	*Vaccinium stamineum* Linneaus	400	2048	1536	930
STMHD4017	Leatherwood	*Dirca palustris* L.	400	2048	1536	930
STMHD0001	Nuttall oak	*Quercus texana* Buckley	100	1024	768	118
STMPP1445	Poplar	*Populus* L.	200	2048	1536	476
STMHD5416	Post oak	*Quercus stellata* Wangenh.	400	2048	1536	930
STMHD4147	Red maple	*Acer rubrum* L.	400	2048	1536	930
STMHD2388	Shagbark hickory	*Carya ovata* (Mill.) K. Koch	200	1024	768	239
STMHD2408	Shumard oak	*Quercus shumardii* Buckley	200	1024	768	239
STMHD6341	Swamp chestnut oak	*Quercus michauxii* Nutt.	400	1024	768	465
STMHD6510	Water oak	*Quercus nigra* L.	400	1024	768	465
STMHD7133	Willow	*Salix* L.	400	2048	1536	930
STMHD3138	Willow oak	*Quercus phellos* L.	200	1024	768	239
STMHD3473	Winged elm	*Ulmus alata* Michx.	200	1024	768	239

*Pixels per 0.1 mm line.

## Technical Validation

**Fig. 3:**
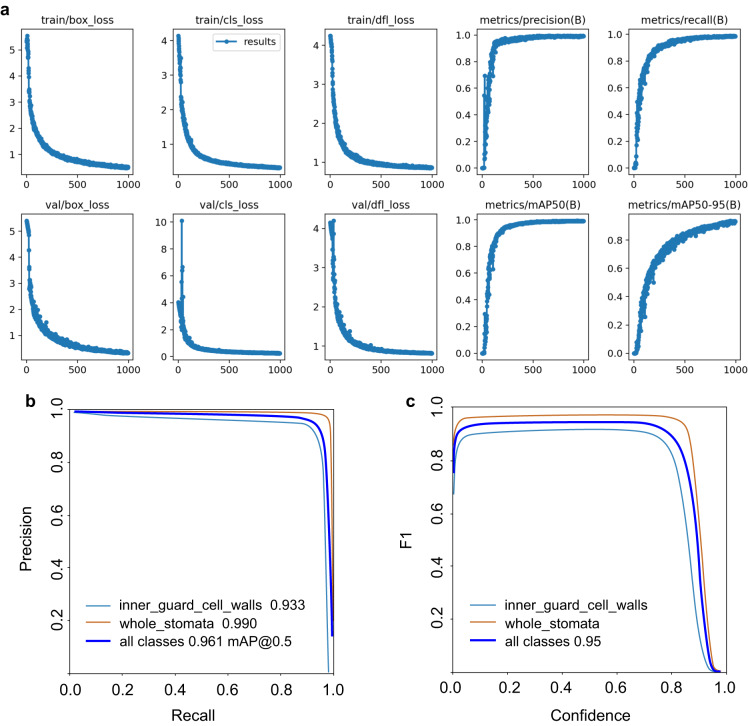
Training and validation results of YOLOv8 models using our hardwood stomatal image dataset **(a)**, and the model performance **(b)** and **(c)**. Train/box_loss, train/cls_loss, train/dfl_loss indicate the bounding boxes loss, class loss, and distribution focal loss, respectively, during the training process; Val/box_loss, val/cls_loss, val/dfl_loss represent the bounding boxes loss, class loss, and distribution focal loss, respectively, during the validation process; metrics/mAP50(B), metrics/recall(B) represent the model evaluation metrics, the mAP50 represents mean average precision at intersection over union (IOU) = 0.50, B is used to distinguish the metrics of segmentation (i.e., metrics/precision(B) for detection and metrics/precision(M) for segmentation).

Images, labels, and data records underwent a rigorous review process to ensure accuracy. The stomatal image dimension (number of pixels in width and height) was verified based on its property information, and the resolution (pixels per 0.1 mm line) was measured and verified using ImageJ software^21^. To assess and validate the quality of images and labels for model training, the dataset was evaluated using YOLOv7 and YOLOv8 models. Figure 3 presents the results obtained from the testing and validation process. To illustrate, we randomly selected 1,123 images for training, and the YOLOv8 models were trained for 993 epochs, and the most optimal model achieved a precision of 0.99168, a recall of 0.98522, a mean average precision at intersection over union (IOU) = 0.50 (mAP@50) of 0.9915, and a mAP@50–95 of 0.9297.

## Usage Notes

To prepare the dataset for object detection model training, we recommend uploading the desired images and labels to Roboflow. This platform can be used to verify and correct annotations, convert existing YOLO annotations to other formats, and perform operations such as resizing, grayscale conversion, auto-orientation, and contrast adjustments. The dataset can also be randomly divided into training, validation, and testing subsets. To create a machine learning model that can be applied to a wider range of species, it is advisable to prepare a training image dataset comprising various species, dimensions, magnifications, and image quality. Including images with diverse quality levels, such as noise (i.e., different color points, stain, and patches), blur, or other imperfections, is also recommended. This will enable the model to learn to identify different stomata of different species even in low-quality images.

**Fig. 4:**
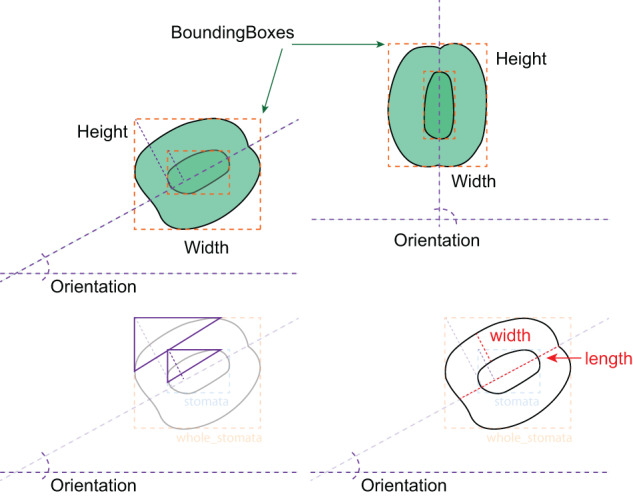
Schematic diagram of measuring leaf stomatal guard cell length and width.

**Fig. 5:**
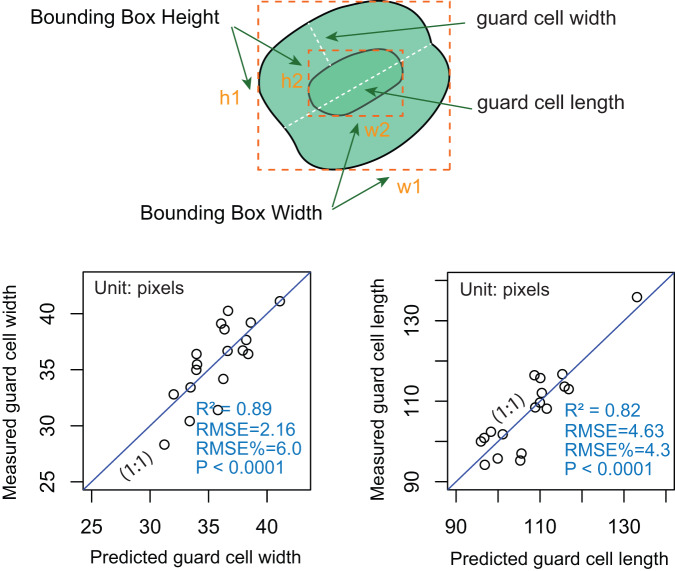
Trained weighted multiple linear regression models to estimate guard cell width and length. Models were trained using bounding boxes’ height and width of *inner_guard_cell_walls* and *whole_stomata* as independent variables.

Once the models are trained, users can extract the detected features and use them to create new indices for assessing stomatal arrangement, operation, and potential functionalities. For instance, the YOLO model’s detected bounding box width and height can be employed for stomatal orientation, estimation of stomatal area, and stomatal area variance^13^. Additionally, regression models can be constructed to estimate other indices, such as leaf stomatal guard cell and aperture width, length, and area, based on the detected bounding box width, height, and/or orientation. A conceptual diagram of this approach is provided in Fig. 4. Specifically, guard cell length is typically defined as the distance between the tips of the two guard cells surrounding the stomatal pore^22^. Therefore, to accurately derive the guard cell length from the output of StoManager1, users may need to incorporate the width, height, and orientation of the *inner_guard_cell_walls* and *whole_stomata*. One possible approach could be to use the orientation information to determine the angle between the two guard cells and then use trigonometry to calculate the guard cell length based on the width and the height of the bounding boxes measurements. Alternatively, users can build the relationships between guard cell length, width, and the bounding boxes’ width, height, and orientation. We also developed two weighted multivariate linear regression models using bounding boxes’ height and width of *inner_guard_cell_walls* and *whole_stomata* as independent variables, which can explain over 81 and 88% variation in measured stomatal guard cell length and width respectively (Fig. 5). Detailed model fitting and plotting can be found in the GitHub repository (https://github.com/JiaxinWang123/ScientificData_Labeled_Hardwood_Images).

## Data Availability

To ensure that the dataset can be easily reproduced and expanded upon in the future, we have made all the Python and R code used to generate and validate the resource available on a code repository (https://github.com/JiaxinWang123/ScientificData_Labeled_Hardwood_Images). StoManager1’s source code and an online demonstration are available on GitHub (https://github.com/JiaxinWang123/StoManager1), along with a user-friendly Windows application on Zenodo^13^.
